# Allergic diseases and asthma in the family predict the persistence and onset-age of asthma: a prospective cohort study

**DOI:** 10.1186/s12931-014-0152-8

**Published:** 2014-11-27

**Authors:** Elina MS Paaso, Maritta S Jaakkola, Aino K Rantala, Timo T Hugg, Jouni JK Jaakkola

**Affiliations:** Center for Environmental and Respiratory Health Research, University of Oulu, PO Box 5000, , FI-90014 Oulu, Finland; Respiratory Medicine Unit, Department of Medicine, Oulu University Hospital, Oulu, Finland; Respiratory Medicine Unit, Institute of Clinical Medicine, University of Oulu, Oulu, Finland; Public Health, Institute of Health Sciences, University of Oulu, Oulu, Finland; Medical Research Center Oulu, University of Oulu, Oulu, Finland

**Keywords:** Asthma, Allergic diseases, Family history, Heredity, Cohort study, Risk ratio, Longitudinal study, Heterogeneity, Epidemiology, Age of onset

## Abstract

**Background:**

Family history of asthma and other allergic diseases have been linked to the risk of childhood asthma previously, but little is known about their effect on the age-of-onset and persistency of asthma until young adulthood.

**Methods:**

We assessed the effect of the family history of asthma and allergic diseases on persistent vs. transient, and early- vs. late-onset persistent asthma in The Espoo Cohort Study 1991–2011, a population-based cohort study of 1623 subjects (follow-up rate 63.2%). The determinants were *any family history* (any parent or sibling); *maternal*; *paternal*; *siblings only*; *parents only*; and *both siblings and parents*. Analyses were conducted separately for asthma and allergic diseases while taking the other disease into account as a confounding factor. The outcomes were *persistent, transient, early-onset persistent *(<13 years) and *late-onset persistent* asthma. Adjusted risk ratios (RR) were calculated applying Poisson regression. Q-statistics were used to assess heterogeneity between RRs.

**Results:**

Family history was associated with the different subtypes but the magnitude of effect varied quantitatively. *Any family history of asthma* was a stronger determinant of persistent (adjusted RR = 2.82, 95% CI 1.99-4.00) than transient asthma (1.65, 1.03-2.65) (heterogeneity: *P* = 0.07) and on early-onset than late-onset persistent asthma. Also *any family history of allergic disease*s was a stronger determinant of persistent and early-onset asthma. The impact of paternal asthma continued to young adulthood (early-onset: 3.33, 1.57-7.06 vs. late-onset 2.04, 0.75-5.52) while the influence of maternal asthma decreased with age (Early-onset 3.94, 2.11-7.36 vs. Late-onset 0.88, 0.28-2.81). Paternal allergic diseases did not follow the pattern of paternal asthma, since they showed no association with late-onset asthma. Also the effect estimates for other subtypes were lower than in other hereditary groups (persistent 1.29, 0.75-2.22 vs. transient 1.20, 0.67-2.15 and early-onset 1.86, 0.95-3.64 vs. late-onset 0.64, 0.22-1.80).

**Conclusions:**

Family history of asthma and allergic diseases are strong determinants of asthma, but the magnitude of effect varies according to the hereditary group so that some subtypes have a stronger hereditary component, and others may be more strongly related to environmental exposures. Our results provide useful information for assessing the prognosis of asthma based on a thorough family history.

## Background

Family history of asthma and allergic diseases have been linked to the risk of childhood asthma [1–3]. In our recent article we showed that family history of asthma is a strong determinant of developing asthma through childhood and into young adulthood [4], and the role of heredity has also been shown to influence adult-onset asthma [5].

Even though asthma is considered a chronic condition, especially children may recover from it [6]. Asthma manifests clinically as different subtypes of disease, including persistent and transient and early and late onset asthma, which may be related to different hereditary patterns [7,8]. Our systematic search identified only two previous longitudinal studies assessing the impact of heredity on the development of different subtypes of asthma, both expanding from birth to 6 years of age [2,8]. These two studies focused on maternal asthma only, and the influence of paternal asthma, siblings’ asthma and the effect of hereditary allergic diseases has only been addressed in cross-sectional studies [1,3,9]. Also, since all of these studies were conducted among children, they focused mostly on childhood wheezing patterns and only few studies applied doctor-diagnosed asthma [1,3].

Hereditary patterns are complex and assessment of their impact requires longitudinal studies expanding from early childhood to adulthood. Also, previous studies on this topic were mostly conducted on small children and therefore assessing the effects on doctor-diagnosed asthma instead of wheezing patterns is difficult. To capture the dynamics of developing asthma and potentially recovering from it in relation to parental and siblings’ asthma and allergic disease, we utilized The Espoo Cohort Study, a population-based 20-year prospective follow-up.

## Methods

### Study population

The source population included all children of the city of Espoo who were born between January, 1984 and March, 1990. A parent-administered baseline questionnaire was distributed in March 1991 to a random sample of children drawn from the roster of Statistics Finland [10]. The baseline population included 2568 children whose parents filled in the questionnaire and in March 1997, a six-year follow-up survey of the cohort was conducted with a follow-up rate of 77.3%. Details of the baseline study population and six-year cohort have been described elsewhere [10,11]. In 2010–11 we conducted the 20-year follow-up with 1623 participants (63.2% of the baseline). The present analyses focused on this 20-year cohort. The study protocol was approved by the Ethics Committee of the Oulu University Hospital District.

### Health outcome

The outcome of interest was the development of asthma from the birth to the end of the study period. In the baseline and the 6-year follow-up the outcome information was based on the question answered by a parent/guardian: “Has the child ever had doctor diagnosed asthma? If yes, at what age did it start?” In the 20-year follow up the study subjects themselves answered the corresponding question.

We defined two categories of asthma according to its dynamics: persistent and transient. Persistent asthma was defined as the presence of asthma with asthmatic symptoms (cough, phlegm production, wheezing, and shortness of breath when not having flu) and/or medication use in all the follow-ups after the first indication of doctor-diagnosed asthma. If asthma was diagnosed between the 6-year and 20-year follow-ups a minimum duration of 5 years was required to categorize the disease as persistent (n = 52). Transient asthma was defined as the presence of asthma at baseline or the 6-year follow-up and lack of asthmatic symptoms and asthma medication in the 20-year follow-up. We excluded 11 subjects with asthma who had experienced a relapse, because they did not represent either the transient or the persistent subgroup of asthmatics thus forming an interesting subtype to be studied further. We also excluded 8 subjects who had had asthma for less than 5 years. Thus, the total population in the present analyses included 1604 subjects. We defined early- and late-onset asthma using a cut-off point of 13 years. There were 40 people with missing information on the onset-age of asthma in the baseline-population of 2568. This information was complemented as described elsewhere [4].

### Definition of asthma and allergic diseases in first-degree relatives

The presence of parental asthma was defined using information from all the three questionnaires. The information on siblings’ asthma status was only asked in the 6- and 20-year follow-ups. We excluded 4 subjects from the sibling asthma analyses due to missing information. The information on parental allergic diseases was required in all three follow-ups whereas siblings’ allergic disease status was only asked in the 20-year follow-up questionnaire. In the baseline and 6-year follow-ups the parental allergic disease status concerned only allergic rhinitis, whereas in the 20-year follow-up the question included allergic rhinitis, allergic conjunctivitis and allergic (atopic) dermatitis without further elaborating which of the different condition(s) were present. 22 subjects were excluded from the siblings’ and 3 from the parental allergic disease analyses due to missing information.

### Covariates

Several potential determinants of asthma were included in the analyses to get the best unbiased estimates of the relation between heredity and the risk of asthma. The information on the covariates was collected from all the three questionnaires. The following variables were adjusted for in the analyses: age [12], sex [13], family socioeconomic status at baseline [14], duration of breast-feeding [15], second-hand smoke exposure at the age of 0 to 3 years [16], mold exposure during the first three years of life [11] and maternal smoking in pregnancy [16]. In the analyses of late-onset persistent asthma the mold and second-hand smoke exposures were taken into account up to the age of 13. The coexistence of hereditary asthma and allergic diseases was taken into account by adjusting for the other disease in the analyses.

### Statistical methods

First, we compared the occurrence of persistent and transient asthma, and early-onset and late-onset persistent asthma according to the following hereditary categories: 1) *any heredity* (either one or both parents and/or any siblings); 2) *maternal* (only mother); 3) *paternal* (only father); 4) *both parents* (both parents) 5) *siblings only* (only siblings, not parents); 6) *parents only* (only parents, not siblings); 7) *both groups* (both siblings and parents) 8) using *no family history* as the reference category. The same categories were formed separately for asthma and allergic diseases.

**Figure 1:**
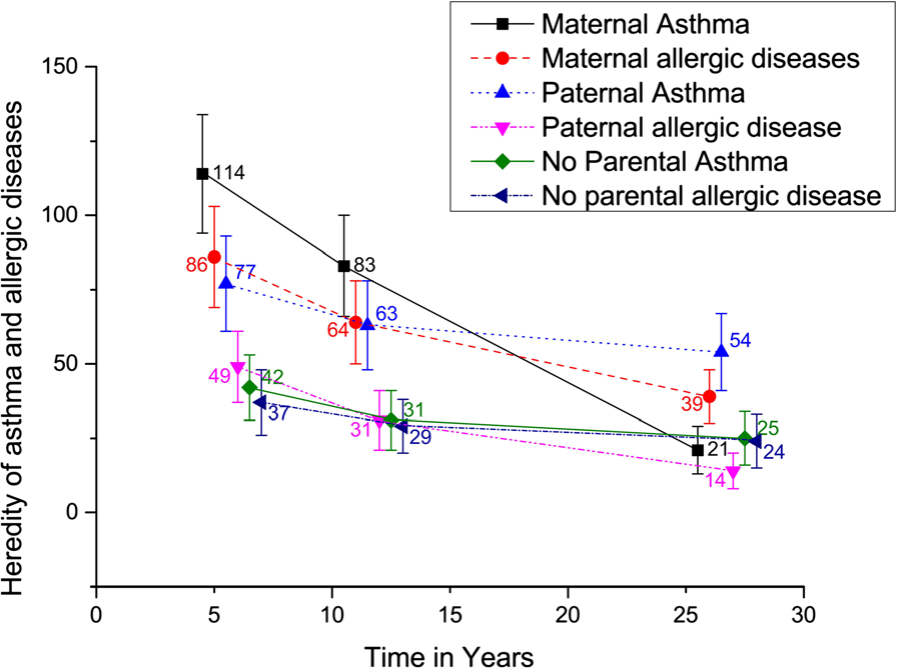
**Incidence rate of persistent asthma per 10 000 person-years according to parental asthma and allergic diseases.** The figure displays the age-specific incidence rates per 10 000 person-years for the age periods of 0 to 6 years, 7 to 12 years, and 13 to 17 years. The numbers displayed are the point estimates of the incidence rates for each hereditary category (maternal asthma: square, maternal allergic diseases: circle, paternal asthma: triangle, paternal allergic diseases: downwards triangle, no parental asthma: diamond, no parental allergic diseases: left-pointing triangle). The vertical lines indicate the 95% confidence intervals calculated by the method of Daly [19].

Second, we quantified the relations between heredity and type of asthma using Poisson regression analysis using the proc genmod –procedure in SAS statistical software package (SAS, version 9.3, SAS Institute, Cary, NC). The resulting RRs were adjusted for potential confounding. Finally, we assessed the difference between adjusted RRs for persistent vs. transient asthma and early- vs. late-onset asthma using Q-statistics for heterogeneity. Heterogeneity Q-statistics were calculated with the derSimonian-Laird –method with a SAS-macro provided by Herzmark and Spiegelman [17,18]. The greater the Q-statistics and the smaller the P-value, the greater the heterogeneity between the studied RRs. The 95% confidence intervals (95% CI) for the incidence rates in Figure 1 were calculated with a SAS macro by Daly [19].

## Results

### Characteristics of the study population

Table 1 presents the characteristics of the baseline, 6-year and 20-year study population. The 20-year cohort used in these analyses did not differ substantially from the baseline study population indicating that selection bias is not an issue in this cohort.

**Table 1:** **Personal and environmental characteristics of the baseline study population, those lost to follow-up, the 6-year cohort and the 20-year cohort, the espoo cohort study 1991–2011, Finland**

	**Baseline**	**Lost to follow-up**	**6-year cohort**	**Lost to follow-up**	**20-year cohort**	**Participation in all surveys**
**No. (% of baseline population)**	**2568 (100.0)**	**584 (22.7)**	**1984 (77.3)**	**945 (36.8)**	**1623 (63.2)**	**1298 (50.5)**
**Age (years)**						
1	424 (16.5)	100 (17.1)	324 (16.3)	156 (16.5)	268 (16.5)	210 (16.2)
2	405 (15.8)	104 (17.8)	301 (15.2)	147 (15.6)	258 (15.9)	199 (15.3)
3	411 (16.0)	92 (15.8)	319 (16.1)	145 (15.3)	266 (16.4)	211 (16.3)
4	400 (15.6)	67 (11.5)	333 (16.8)	159 (16.8)	241 (14.9)	209 (16.1)
5	415 (16.2)	101 (17.3)	314 (15.8)	151 (16.0)	264 (16.3)	208 (16.0)
6-7	513 (20.0)	120 (20.6)	393 (19.8)	187 (19.8)	326 (20.1)	261 (20.1)
**Sex**						
male	1311 (51.1)	309 (52.9)	1002 (50.5)	557 (58.9)	754 (46.5)	592 (45.6)
female	1257 (49.0)	275 (47.1)	982 (49.5)	388 (41.1)	869 (53.5)	706 (54.4)
**Family socioeconomic status at baseline**						
low	667 (26.1)	169 (29.1)	498 (25.2)	296 (31.5)	371 (22.9)	292 (22.5)
middle/high	1889 (73.9)	411 (70.9)	1478 (74.8)	643 (68.5)	1246 (77.1)	1003 (77.5)
**Duration of breast-feeding in months**						
< 4	481 (19.3)	135 (24.1)	346 (17.9)	192 (21.0)	289 (18.3)	221 (17.5)
4 ≤ x < 8	670 (26.9)	159 (28.3)	511 (26.4)	250 (27.4)	420 (26.6)	333 (26.3)
≥ 8	1343 (53.9)	267 (47.6)	1076 (55.7)	471 (51.6)	872 (55.2)	711 (56.2)
**SHS**_**0–3**_						
yes	299 (11.6)	66 (11.3)	233 (11.7)	105 (11.1)	194 (12.0)	153 (11.8)
no	2269 (88.4)	518 (88.7)	1751 (88.3)	840 (88.9)	1429 (88.1)	1145 (88.2)
**Mold**_**0–3**_						
yes	138 (5.4)	21 (3.6)	117 (5.9)	55 (5.8)	83 (5.1)	73 (5.6)
no	2428 (94.6)	569 (96.4)	1867 (94.1)	888 (94.2)	1540 (94.9)	1225 (94.4)
**SHS**_0–13_^*^						
yes	468 (18.2)	121 (20.7)	347 (17.5)	161 (17.0)	307 (18.9)	234 (18.0)
no	2100 (81.8)	463 (79.3)	1637 (82.5)	784 (83.0)	1316 (81.1)	1064 (82.0)
**Mold**_0–13_^*^						
yes	385 (15.0)	35 (6.0)	350 (17.6)	150 (15.9)	235 (14.5)	220 (17.0)
no	2183 (85.0)	549 (94.0)	1634 (82.4)	795 (84.1)	1388 (85.5)	1078 (83.1)
**Maternal smoking in pregnancy**						
yes	364 (14.2)	104 (17.9)	260 (13.1)	160 (17.0)	204 (12.6)	156 (12.0)
no	2199 (85.8)	477 (82.1)	1722 (86.9)	784 (83.1)	1415 (87.4)	1141 (88.0)

Abbreviations: SHS_0–3_ = exposure to second-hand smoke at the age of 0–3, SHS_0–13_ = exposure to second-hand smoke at the age of 0–13.

Missing information: 12 for family socioeconomic status at baseline, 74 for duration of breast-feeding, 5 for maternal smoking in pregnancy, 2 from mold-exposure from 0 to 3 years.

*Used for adjustment when studying late-onset asthma as the outcome.

The occurrence of persistent, early- and late-onset persistent and transient asthma and the percentage of subjects without any asthma according to the different categories of hereditary asthma and allergic diseases are shown in Table 2. The overall incidence rates of asthma per 10 000 person-years were 70.7 (95% CI 55.2-89.2) for asthma in general, 39.9 (28.5-54.3) for persistent asthma, 25.6 (16.7-37.6) for transient asthma, 26.2 (17.1-38.3) for early-onset asthma and 13.7 (7.4-23.1) for late-onset asthma.

**Table 2:** **The prevalences of different subtypes of asthma according to hereditary groups, the espoo cohort study 1991–2011, Finland**

	**Persistent asthma N (%)**		**Transient asthma N (%)**	**No asthma ever N (%)**	**Total**
	**Early-onset N (%)**	**Late-onset N (%)**			
Total	134 (8.4)		86 (5.4)	1384 (86.3)	1604 (100.0)
	88 (5.5)	46 (2.9)			
No family history of asthma	73 (6.2)		57 (4.8)	1046 (88.9)	1176 (73.4)
	42 (3.6)	31 (2.6)			
No family history of allergic diseases	33 (5.2)		35 (5.5)	564 (89.2)	632 (39.4)
	18 (2.8)	15 (2.4)			
Any family history of asthma	61 (14.4)		29 (6.8)	335 (78.8)	425 (26.5)
	46 (10.8)	15 (3.5)			
Any family history of allergic diseases	100 (10.5)		50 (5.3)	800 (84.2)	950 (59.3)
	70 (7.4)	30 (3.2)			
Maternal asthma	20 (12.9)		12 (7.7)	123 (79.4)	155 (9.7)
	17 (11.0)	3 (1.9)^a^			
Maternal allergic diseases	40 (12.4)		22 (6.8)	260 (80.7)	322 (20.1)
	28 (8.7)	12 (3.7)			
Paternal asthma	15 (13.5)		5 (4.5)^a^	91 (82.0)	111 (6.9)
	9 (8.1)	6 (5.4)			
Paternal allergic diseases	17 (6.2)		15 (5.5)	243 (88.4)	275 (17.1)
	13 (4.7)	4 (1.5)^a^			
Both parents with asthma	8 (36.4)		2 (9.1)^a^	12 (54.5)	22 (1.4)
	5 (22.7)	3 (13.6)^a^			
Both parents with allergic diseases	21 (16.8)		6 (4.8)	98 (78.4)	125 (7.8)
	14 (11.2)	7 (5.6)			
Only siblings with asthma	18 (13.1)		10 (7.3)	109 (79.6)	137 (8.5)
	15 (10.9)	3 (2.2)^a^			
Only siblings with allergic diseases	22 (9.4)		7 (3.0)	206 (87.7)	235 (14.7)
	15 (6.4)	7 (3.0)			
Only parents with asthma	27 (11.8)		16 (7.0)	185 (81.1)	228 (14.2)
	19 (8.3)	8 (3.5)			
Only parents with allergic diseases	38 (8.6)		29 (6.5)	377 (84.9)	444 (27.7)
	26 (5.9)	12 (2.7)			
Both siblings and parents with asthma	16 (27.1)		3 (5.1)^a^	40 (67.8)	59 (3.7)
	12 (20.3)	4 (6.8)^a^			
Both siblings and parents with allergic diseases	40 (14.8)		14 (5.2)	217 (80.1)	271 (16.9)
	29 (10.7)	11 (4.1)			

% is the percentage of people that represent the corresponding subtype of asthma divided by the total number of people that have the risk factor mentioned in that line (e.g. maternal asthma, paternal allergic diseases, total n in the group mentioned in the last column of the table).

^a^Restricted amount of observations in the cell may lead to chance variation in the results.

19 people excluded from the analyses due to either asthma relapse (N = 11) or follow-up time of less than 5 years (N = 8).

Missing information: 1 in parental asthma, 4 for siblings with asthma, 3 in parental allergic diseases, 22 in siblings with allergic diseases.

### Transient versus persistent asthma

Table 3 shows that heredity has a stronger impact on the risk of persistent asthma compared to transient asthma. In the group of *any family history of asthma*, the adjusted RR for persistent asthma was 2.82, 95% CI 1.99-4.00, whereas the RR of developing transient asthma was 1.65, 95% CI 1.03-2.65. The Q-statistics indicated heterogeneity between the effect estimates (Q = 3.20, *P* = 0.07). For *any family history of allergic diseases* the adjusted RR for persistent asthma was 2.20, with 95% CI 1.48-3.28 and for transient asthma was 1.02 (0.66-1.58) (Q = 6.50, *P* = 0.01). The other hereditary groups showed smaller effect estimates for developing transient asthma in comparison to higher estimates for developing persistent asthma (Table 3). An exception to this was seen with paternal allergic diseases where both the RR for developing persistent asthma as well as the RR for transient asthma were virtually equal and non-significant (Persistent asthma: 1.29, 0.75-2.22; Transient asthma: 1.20, 0.67-2.15; heterogeneity Q = 0.03, *P =* 0.86).

**Table 3:** **The influence of family history of asthma on persistent and transient asthma, The Espoo Cohort Study 1991–2011, Finland**

	**Persistent asthma vs. no asthma**		**Transient asthma vs. no asthma**		**Heterogeneity** ^**b**^
**Family history of asthma**	**RR**	**RR** ^**a**^	**RR**	**RR** ^**a**^	**Q-statistic,**
	**(95% CI)**	**(95% CI)**	**(95% CI)**	**(95% CI)**	***P*** **-value**
*Parents or siblings with asthma*	2.62 (1.88-3.65)	2.82 (1.99-4.00)	1.56 (0.99-2.43)	1.65 (1.03-2.65)	3.20, 0.07
*Parents or siblings with allergic diseases*	2.11 (1.45-3.09)	2.20 (1.48-3.28)	0.98 (0.65-1.49)	1.02 (0.66-1.58)	6.50, 0.01
*Maternal asthma*	2.42 (1.47-3.98)	2.50 (1.50-4.15)	1.64 (0.89-3.04)	1.57 (0.79-3.11)	1.35, 0.25
*Maternal allergic disease*	2.31 (1.54-3.46)	2.17 (1.40-3.37)	1.44 (0.88-2.38)	1.48 (0.87-2.52)	1.18, 0.77
*Paternal asthma*	2.47 (1.43-4.28)	2.70 (1.52-4.80)	0.97 (0.39-2.40)	1.10 (0.44-2.73)	0.85, 0.35
*Paternal allergic disease*	1.13 (0.66-1.94)	1.29 (0.75-2.22)	1.07 (0.61-1.91)	1.20 (0.67-2.15)	0.03, 0.86
*Both parents with asthma*	6.92 (3.76-12.71)	6.70 (3.34-13.45)	2.64 (0.71-9.86)	2.94 (0.80-10.87)	4.24, 0.04
*Both parents with allergic disease*	3.05 (1.88-4.95)	3.32 (2.01-5.47)	1.07 (0.46-2.45)	0.97 (0.39-2.40)	5.41, 0.02
*Siblings with asthma only*	2.11 (1.31-3.40)	2.33 (1.42-3.84)	1.62 (0.85-3.09)	1.79 (0.93-3.45)	7.36, 0.01
*Siblings with allergic disease only*	1.67 (0.99-2.80)	1.75 (1.02-3.01)	0.57 (0.26-1.27)	0.63 (0.28-1.41)	4.27, 0.04
*Parents with asthma only*	2.41 (1.52-3.84)	2.50 (1.54-4.06)	1.55 (0.88-2.73)	1.56 (0.85-2.87)	4.29, 0.04
*Parents with allergic disease only*	1.84 (1.16-2.92)	1.82 (1.12-2.97)	1.19 (0.74-1.91)	1.23 (0.75-2.03)	1.20, 0.27
*Parents and siblings with asthma*	5.52 (3.31-9.20)	5.97 (3.48-10.22)	1.39 (0.45-4.32)	1.64 (0.52-5.14)	17.40, 0.00
*Parents and siblings with allergic disease*	3.13 (2.00-4.90)	3.48 (2.18-5.56)	1.01 (0.55-1.84)	1.02 (0.54-1.92)	9.40, 0.00

19 People excluded from the analyses due to follow-up period of less than 5 years (n = 8) or asthma relapse (n = 11).

^a^Adjusted for sex, age, family socioeconomic status at baseline, maternal smoking in pregnancy, SHS at the age of 0–3, mold exposure at the age of 0–3, and duration of breast-feeding.

^b^Heterogeneity calculated between the adjusted risk ratios of persistent and transient asthma.

### Early- versus late-onset persistent asthma

Heredity plays the most important role as the determinant of the early-onset persistent asthma, using 13 years of age as the cut-off point (Table 4). Almost all the effects of the hereditary determinants showed substantial heterogeneity between early- and late-onset asthma. The strongest risk for developing early-onset asthma was detected in the hereditary group *both parents with asthma* where the adjusted RR was 9.61 (95% CI 3.93-23.45), and *both parents and siblings* having asthma. With family history of allergic diseases these were the same groups with highest RRs. (Table 4) Interestingly, increased risk related to paternal asthma extended to late-onset persistent asthma (adjusted RR 2.04, 95% CI 0.75-5.52), but the effect related to maternal asthma diminished with age (adjusted RR 0.88, 95% CI 0.28-2.81). This pattern was not replicated with *paternal allergic disease*, which showed some effect on early-onset persistent asthma (adjusted RR 1.86, 95% CI = 0.95-3.64) but null effect on late-onset persistent asthma (adjusted RR 0.64, 95% CI 0.22-1.80). To highlight the difference between maternal and paternal asthma and allergic diseases on the development of childhood asthma we also calculated age-specific incidence rates from 0–6 years of age, 6–12 years of age and 13–27 years of age. (Figure 1). The incidence rate of persistent asthma related to no parental asthma decreases only slightly over time while the incidence rate of persistent asthma related to maternal asthma decreases significantly over time. Also the incidence rates of maternal and paternal allergic diseases decrease with time while the effect of paternal asthma on the incidence rate of persistent asthma remains nearly constant through the age periods.

**Table 4:** **The influence of family history of asthma and allergic diseases on early- and late-onset persistent asthma, The Espoo Cohort Study 1991–2011, Finland**

	**Early onset persistent asthma vs. no asthma**		**Late onset persistent asthma vs. no asthma**		**Heterogeneity** ^**c**^
**Family history of asthma**	**RR**	**RR** ^**a**^	**RR**	**RR** ^**b**^	**(Q-statistic,**
	**(95% CI)**	**(95% CI)**	**(95% CI)**	**(95% CI)**	***P*** **-value)**
*Parents or siblings with asthma*	3.58 (2.37-5.41)	4.10 (2.66-6.32)	1.57 (0.84-2.92)	1.44 (0.73-2.84)	6.47, 0.01
*Parents or siblings with allergic diseases*	2.79 (1.70-4.60)	3.24 (1.89-5.58)	1.43 (0.77-2.67)	1.26 (0.67-2.38)	4.94, 0.03
*Maternal asthma*	3.60 (2.00-6.50)	3.94 (2.11-7.36)	0.92 (0.28-3.07)	0.88 (0.28-2.81)	4.98, 0.03
*Maternal allergic disease*	2.89 (1.71-4.86)	3.04 (1.72-5.35)	1.71 (0.84-3.47)	1.27 (0.58-2.80)	3.07, 0.08
*Paternal asthma*	2.70 (1.30-5.61)	3.33 (1.57-7.06)	2.42 (0.99-5.91)	2.04 (0.75-5.52)	0.60, 0.44
*Paternal allergic disease*	1.51 (0.78-2.90)	1.86 (0.95-3.64)	0.63 (0.22-1.83)	0.64 (0.22-1.80)	2.89, 0.09
*Both parents with asthma*	8.73 (3.80-20.04)	9.61 (3.93-23.45)	7.75 (2.57-23.39)	5.46 (1.30-22.98)	0.42, 0.51
*Both parents with allergic disease*	3.71 (1.99-6.92)	4.48 (2.35-8.57)	2.58 (1.11-5.99)	2.41 (0.98-5.94)	1.20, 0.27
*Siblings with asthma only*	2.96 (1.70-5.15)	3.30 (1.84-5.92)	0.95 (0.30-3.04)	1.10 (0.33-3.61)	4.44, 0.04
*Siblings with allergic disease only*	2.08 (1.06-4.08)	2.31 (1.11-4.78)	1.24 (0.52-2.96)	1.24 (0.52-2.99)	1.13, 0.29
*Parents with asthma only*	3.24 (1.79-5.86)	3.89 (2.10-7.20)	1.60 (0.70-3.62)	1.21 (0.49-2.96)	2.64, 0.10
*Parents with allergic disease only*	2.43 (1.34-4.41)	2.85 (1.51-5.39)	1.25 (0.58-2.71)	0.92 (0.40-2.13)	4.41, 0.04
*Parents and siblings with asthma*	8.19 (4.31-15.57)	9.24 (4.68-18.22)	3.58 (1.27-10.14)	3.38 (1.13-10.18)	2.31, 0.13
*Parents and siblings with allergic disease*	4.44 (2.49-7.92)	5.50 (2.95-10.24)	1.96 (0.89-4.29)	1.93 (0.87-4.30)	3.36, 0.02

19 People excluded from the analyses due to follow-up period of less than 5 years (n = 8) or asthma relapse (n = 11).

^a^Adjusted for sex, age, family socioeconomic status at baseline,

maternal smoking in pregnancy, SHS at the age of 0–3, mold exposure at the age of 0–3, and duration of breast-feeding.

^b^Adjusted for sex, age, family socioeconomic status at baseline,

maternal smoking in pregnancy, SHS from birth to 13 years of age, duration of breast-feeding, mold exposure from birth to the age of 13.

^c^Heterogeneity calculated between the adjusted risk ratios of early-onset and late-onset persistent asthma.

## Discussion

### Main findings

This population-based prospective 20-year cohort study provides evidence that hereditary asthma and allergic diseases are consistently determinants of different subtypes of asthma, especially persistent rather than transient and early-onset rather than late-onset persistent asthma. The effect related to paternal asthma seemed to remain more constant over time as that of maternal asthma as shown in Figure 1. Both of these findings are consistent with the results of a Finnish population-based case–control study of adult-onset asthma, where paternal asthma was shown to have a stronger effect of adult onset asthma compared with maternal asthma [5]. Interestingly, paternal allergic diseases didn’t seem to follow this pattern and showed small, non-significant effects on all of the subtypes studied with early-onset persistent asthma being the only subtype with an elevated risk ratio. (Figure 1; Tables 3 and 4).

### Validity of results

According to our systematic literature search, the current study has the longest follow-up of the studies concerning heredity and development and dynamics of asthma. This enabled us to assess the role of hereditary asthma in both early- versus late-onset as well as persistent versus transient asthma up to adulthood. As shown in Table 1, the baseline - and the 20-year follow-up populations were similar, which assures that selection bias is not an issue in this study.

The definition of asthma was based on parent- or self-reported doctor-diagnosed asthma. Due to the national health care system the access to medical care has excellent coverage in Finland, and the special reimbursement provided nationally for asthma medications provides an economic incentive for actually having potential asthma diagnosed by a physician. These factors are likely to reduce misclassification of asthma in our study population. In addition, according to the study of Pattaro et al. utilizing the age of asthma onset in the analyses improves the validity of the estimate of the incidence of asthma [20]. Most of the previous studies on this subject focus on childhood wheezing patterns, since the diagnosis of asthma is difficult among children [2,7–9]. In our study, the participants have been followed for 20 years and thus making verification of their asthma diagnosis easier especially for more persistent type of asthma. Also, the classification of persistent asthma required both a positive answer to the question on asthma diagnosis as well as symptoms or medication use, making the diagnosis even stronger.

Having information on the age of onset enabled us to categorize the asthma subtype into early- and late-onset asthma. We used the cut-off point of 13 years for this, which is consistent with a previous study suggesting that asthma starting earlier than 12 years forms a different phenotype [21]. The categorization into transient and persistent asthma was based on the diagnosis of asthma in combination with information on asthma symptoms and/or medication use, which were also collected with the questionnaire.

As sensitivity analyses, we also used eight years as the cut-off point, which resulted in the following crude effect estimates: early-onset: *maternal asthma* 2.87, 95% CI 1.54-5.36, and *paternal asthma* 1.72, 95% CI 0.63-4.68; late-onset: *maternal asthma* 1.47, 95% CI 0.71-3.03, and *paternal asthma* 2.42, 95% CI 1.19-4.89. The same risk ratios for *maternal allergic disease* was for early-onset asthma 3.36, 95% CI 1.64-6.87 and for late-onset asthma 2.59, 95% CI 1.37-4.91 and for *paternal allergic disease* early-onset asthma adjusted RR = 2.16, 95% CI 0.97-4.78 and late-onset 0.95, 95% CI 0.37-2.47. This strengthens the evidence that the influence of paternal asthma continues also after childhood as seen in our analyses in Table 4 and that when studying younger age categories, maternal effect is even stronger than with our cut-off of 13 years.

We also had the possibility to take into account several potential confounding factors. In order to ensure the relevance of confounders, we focused on early-life factors and exposures that took place before the onset of asthma.

### Synthesis with previous knowledge

Our finding of the importance of maternal asthma for early-onset persistent asthma is consistent with the results from the two previous 6-year longitudinal studies [2,8] as well as from the two cross-sectional studies [1,9]. The Tasmanian Longitudinal Health Study provides complementary evidence of the role of maternal asthma in reducing experience of remission in adulthood [22].

London et al. reported that siblings with asthma predict different clinical types of asthma [1]. However, contrary to their results we found that also siblings strongly predicted persistent asthma and early-onset persistent asthma and not late-onset persistent asthma. We also found an elevated risk ratio between siblings with asthma and transient asthma which is partially reflected in the results of London et al. in that they found an association between siblings with asthma and wheeze and asthma-like illness without diagnoses which might be close to our subgroup of transient asthmatics [1].

In one previous study the risk factors of transient wheeze were studied during the first 4 years of life. Also here it was shown that parental asthma and allergic eczema and allergic rhinitis were not related to transient wheezing [23]. This study strengthens the hypothesis, that transient asthma is more related to environmental exposures, since they found e.g. that maternal active smoking in pregnancy and childhood day care center attendance generated a risk for transient wheeze.

As in the studies of London et al. and Rusconi et al. we found that hereditary allergic diseases also play a role in the development of the different phenotypes of asthma [1,9]. However, contrary to their results we didn’t find an association between parental allergic disease and late-onset asthma. This is partly due to our cut-off point between early- and late-onset asthma.

As our study covered the age ranges up to 27 years, our choice of the cut-off point is supported by the study of Miranda et al. finding that children with asthma onset before the age of 12 years have more allergen sensitivity and allergic symptoms than those with a later onset, so this could form its own phenotype [21]. The differences in hereditary patterns related to early- and late-onset persistent asthma could be partly due to different inflammatory mechanisms, as suggested by the results of Miranda et al.

Our study suggests that both paternal and maternal asthma are related to personal asthma of the child. The results concerning paternal asthma are consistent with a recent population-based case–control study on adult-onset asthma from Finland [5], but contrary to some studies focusing only on childhood asthma, where paternal asthma showed either no significant effect on small children’s asthma [3] or was not analyzed [2,8].

Additionally, our study provides evidence that the effect of paternal asthma continues longer than that of maternal asthma (Figure 1). One study conducted on children has also found an impact of paternal asthma on late-onset wheezing when looking at children 6–7 years of age and new-onset wheezing in the previous 12 months [9]. Our findings are in line with a hypothesis that gene-environment interaction is more prominently related to paternally inherited genes. A recent review proposed that maternal influence on IgE-levels of the child begins from antenatal stage and lasts through childhood, while paternal effect on IgE begins later in childhood and increases later on [24]. However, this gives no explanation to the fact that the influence of paternal allergic diseases doesn’t continue to young adulthood underlining the complexity of hereditary patterns in asthma and allergic diseases.

## Conclusions

The results of this population-based 20-year prospective cohort study provide evidence that different subtypes of asthma have different hereditary patterns. While family history of asthma and allergic diseases increase overall the risk of asthma through childhood and young adulthood, the strongest effect of family history of asthma and allergic diseases is seen on the subgroups of persistent- and early-onset asthma. It seems that some subtypes are more correlated with environmental exposures and that some of the impact of the family history of asthma might also be preventable by avoiding environmental factors. Our results suggest that gene-environment interactions need further investigation. For clinicians, they strengthen the evidence that in assessing the patient’s prognosis and altogether the risk of asthma subtypes, a thorough family history of asthma and allergic diseases is required.

### Consent

Written informed consent was obtained from the patient for the publication of this report and any accompanying images.

## Abbreviations

SHS: Second-hand smoke exposure
RR: Risk ratio
95% CI: 95 percent confidence interval
